# Prediction of outcome and selection of the liver transplantat candidate in acute liver failure

**DOI:** 10.3389/fphys.2012.00340

**Published:** 2012-08-28

**Authors:** Johannes Hadem, Christian P. Strassburg, Michael P. Manns

**Affiliations:** ^1^Department of Gastroenterology, Hepatology, and Endocrinology, Medical School HannoverHannover, Germany; ^2^Medical Clinic I – General Internal Medicine, Gastroenterology, Hepatology, Nephrology, Infectious Diseases, and Endocrinology, University Clinic BonnBonn, Germany

**Keywords:** acute hepatitis, biomarker, emergency liver transplantation, hepatic encephalopathy, hepatic failure, outcome, prognosis

## Abstract

Acute liver failure (ALF) is characterized by a sudden and severe deterioration of liver function, typically mirrored by a marked increase of the international normalized ratio (INR) and hepatic encephalopathy (HE). Due to various possible causes hepatocytes get damaged via either apoptotic or necrotic pathways. Anticipating the natural prognosis of a patient with ALF is one of the most challenging tasks in hepatology critical care. Important factors that influence the chance of spontaneous recovery are the underlying etiology of acute liver failure, the acuity of disease, and the severity of HE. Once an estimation of the prognosis in the individual patient has been made, this quickly has to be integrated in the discussion whether high-urgency liver transplantation is necessary and justifiable. This decision has to cover several medical, social, and organizational issues. Well organized liver transplantation programs around the world have achieved an impressive improvement of the 1 year survival rate in ALF from around 40% without transplantation up to nearly 80% with transplantation. The recent debate on whether severe acute alcoholic hepatitis could represent a new candidate eligible for high-urgency liver transplantation shows that the topic is still open for discussion.

## Introduction

The overall incidence of acute liver failure (ALF) in developed countries is estimated to be 1–6 per million people every year (Bower et al., 2007; Bernal et al., 2010). Emergency liver transplantation (ELT) is a potentially life-saving procedure in patients with ALF, but carries relevant long-term morbidity and mortality. Appropriate selection of patients for ELT therefore requires tools allowing to accurately prognosticate spontaneous recovery (Renner, 2007). The prediction of outcome in an individual patient with ALF is among the most challenging tasks in hepatology critical care. Many retrospective observational studies have tried to identify particular clinical and laboratory markers or prognostic models capable of predicting prognosis and need of ELT. This article addresses some of the questions that arise when facing a patient who progressively deteriorates from severe acute hepatitis to ALF.

## Acute or acute-on-chronic—difficult to distinguish

A deteriorating liver function that comes to attention for the first time often triggers the assumption of an ALF. But by definition, ALF implies the absence of chronic liver disease (Trey and Davidson, 1970; Bernal et al., 2010; Lee et al., 2011), and the majority of patients that present with worsening liver function suffer from acute-on-chronic liver failure (i.e., an acute deterioration superimposed on chronic liver disease). Non-alcoholic and alcoholic fatty liver disease with varying degrees of fibrosis are among the most common causes of pre-existing liver injury in those patients. Patients with acute-on-chronic liver failure can often be identified by thorough history taking and physical examination. The role of abdominal ultrasound is more complex. Undoubtedly, ultrasound will detect overt liver cirrhosis presenting with reduced liver volume, irregular liver surface, distorted liver veins, and nodular liver parenchyma (Caselitz, 1999). But cirrhotic parenchymal changes can be difficult to detect in steatosis, and signs of portal hypertension may also develop in subacute liver failure. Furthermore, acute hepatitis triggers inflammatory infiltration, hepatocyte swelling, and early regenerative fibrotic parenchymal changes. These processes have been shown to increase liver stiffness when measured by transient elastography and newer ultrasound technologies and thereby can hamper the distinction between acute and chronic liver injury (Arena et al., 2008; Sagir et al., 2008; Dechêne et al., 2010).

A transjugular liver biopsy might aid in confirming certain ALF etiologies (Lee et al., 2011) and even in defining the degree of liver necrosis (Beckmann et al., 2009). But liver biopsy is not recommended and probably of minor help in distinguishing acute-on-chronic from acute liver failure. Moreover, it remains an open question which degree of pre-existing liver injury should be regarded as a contraindication for ELT. Whereas a significant chronic component of deteriorated liver function is generally regarded a contraindication for ELT, there are at least three exceptions to this rule: ALF in autoimmune hepatitis, Wilson's disease, and Budd-Chiari syndrome can arise from a relapsing or chronic course of disease. Those patients are suitable ELT candidates despite cirrhotic hepatic changes.

## Role of hepatic encephalopathy and etiology in outcome prediction

Not too surprisingly, the prospective observational study by the US ALF Study Group underlined the major prognostic role of hepatic encephalopathy (HE) and etiology in ALF. HE grade possibly reflects the degree of cerebral edema. Whereas ALF patients with a baseline HE grade 1/2 had a transplant-free 21-day survival of 52%, this was only 33% in patients initially presenting with HE grade 3/4 (Ostapowicz et al., 2002). A hyperacute course (interval from icterus to encephalopathy <7 days) of liver failure (O'Grady et al., 1989) that is typically seen in acetaminophen, hepatitis A or herpes simplex virus (Graham et al., 2009; Bernal et al., 2010) is associated with a sudden rise in international normalized ratio (INR), an increased risk of cerebral edema and a rather high transplant-free survival rate. A subacute course (interval from icterus to encephalopathy >21 days) (O'Grady et al., 1989), as seen in indeterminate ALF implies a low transplant-free survival rate despite initially moderate increases in INR and low rates of cerebral herniation (Ostapowicz et al., 2002; Hadem et al., 2008; Bernal et al., 2010).

Data from Europe and the US ALF Study Group show that transplant-free survival rates in ALF patients are strongly associated with the underlying ALF etiology: 60–80% in acetaminophen-induced ALF, 30–40% in severe acute hepatitis B, 10–20% in indeterminate ALF, 20–50% in non-acetaminophen drug-induced ALF, 0–20% in Wilson's disease and around 70–80% in amanita ingestion (Ostapowicz et al., 2002; Escudié et al., 2007; Hadem et al., 2008). Only 10% of patients with drug-induced hepatitis progress to ALF which then implies a bad prognosis even after the provoking medication has been stopped (Andrade et al., 2005; Reuben et al., 2010). Phenprocoumon, a coumadin derivate used in Germany can cause drug-induced ALF associated with low transplant-free survival rates (Hadem et al., 2008; Canbay et al., 2009). Mortality rate in ALF caused by hepatitis E is assumed to be 8–11% in Western countries, but can be as high as 50% when ELT is not available, and may also be higher when acquired during pregnancy (Bernuau et al., 2008). Pregnancy-related ALF is a heterogeneous disease entity which has not sufficiently been studied. A recent observational study reported on 54 patients (18 acute fatty liver of pregnancy, 32 eclampsia-associated) of whom 13% died and 7% received ELT (Westbrook et al., 2010). Budd-Chiari syndrome and early graft dysfunction are among other causes of ALF with high risk of a fatal outcome. Although transjugular intrahepatic portosystemic shunting (TIPS) has undoubtedly decreased the case fatality rate of Budd-Chiari syndrome presenting with ALF, data on the use of TIPS in this setting are still scarce. In the important paper on TIPS in Budd-Chiari syndrome by Garcia-Pagan et al. only 9/124 (7%) had ALF prior to TIPS, only 27/124 (22%) received TIPS due to (acute or chronic) liver failure, and a significant proportion of these patients needed ELT or died (Garcia-Pagán et al., 2008).

## Prognostic markers in acute liver failure

### Rationale behind prognostic markers

Many prognostic markers have been proposed to overcome the prognostic uncertainty that is a major problem in the early phase of ALF. One common approach is to deduce the outcome from the degree of aminotransferase increase. These enzymes are—like many other components of the hepatocellular content—released into the circulation in apoptosis and necrosis. However, there is no reliable association between aminotransferases and outcome. Ischemic hepatitis and acetaminophen toxicity regularly present with huge aminotransferase increases, but often are associated with favorable prognosis. Another approach is to measure serum markers of liver regeneration (e.g., alpha-fetoprotein, cytokines, growth factors, and also phosphate) to get an idea of the hepatic regenerative capacity. These parameters have mainly been studied in patients with hyperacute liver failure in the setting of acetaminophen toxicity. A reasonable way to quantify the severity of liver damage is to extrapolate the course of ALF according to serial hepatocellular function measurements. Synthetic liver capacity is best observed by tracking the INR that reflects the synthesis of clotting factors II, V, VII, and X. Rapid deteriorations can be recognized by decreases of factor V due to its short half time of 12–15 h. Detoxification liver capacity can be monitored by bilirubin, ammonia, indocyanin green, and other serum parameters. Many of these parameters have been incorporated in a number of prognostic criteria in an attempt to predict ALF outcome as accurately as possible.

### Alpha-fetoprotein

Among the early investigations on prognostic markers in ALF is that of Murray-Lyon et al. from King's College Hospital. Sixty-four patients with fulminant hepatic failure were examined regarding their serum alpha-fetoprotein (AFP) levels. AFP levels >50 ng/ml were observed in 48% of the 23 survivors, but only in 10% of the 41 ALF patients with a fatal outcome (Murray-Lyon et al., 1976). In a subsequent study on 239 patients with acetaminophen intoxication, a threshold AFP of ≥3.9 μg/l on day + 1 after peak ALT to identify nonsurvivors had a sensitivity of 100% and a specificity of 74% (Schmidt and Dalhoff, 2005). A retrospective analysis of 206 patients from the US ALF study (80 acetaminophen, 30 indeterminate) showed that an AFP day 3-to-day1 ratio >1 predicted survival with a sensitivity of 65% and a specificity of 84% (Schiødt et al., 2006).

### Lactate

Arterial blood lactate levels can be measured rapidly by point-of-care testing and reflect both increased production from peripheral tissues and the injured liver and reduced clearance from the circulation as a consequence of impaired hepatic metabolic capacity (Bernal, 2010). In a mixed prospective/retrospective study of over 200 patients with acetaminophen-induced ALF, high blood lactate levels were found to be closely related to a fatal outcome (Bernal et al., 2002). Subsequent studies found arterial lactate (baseline or 12 h after admission) to be associated strongly and independently with death or transplantation in both acetaminophen and non-acetaminophen-induced ALF (Macquillan et al., 2005; Schmidt and Larsen, 2006; Hadem et al., 2008; Bernal et al., 2010). Baseline lactate levels were higher (median 4.7 vs. 2.9 mmol/L) and steadily increased in ALF patients with an unfavorable outcome (Hadem et al., 2008). Others have argued that lactate lacks the specificity required to a sole criterion for ELT listing (Schmidt and Larsen, 2010). Different views on the prognostic value of lactate levels in ALF are likely to reflect variability in the timing and degree of fluid resuscitation in early ALF management. The greatest prognostic value will therefore be gained from those patients in whom no correction of initially high levels occurs following volume resuscitation, and these patients should probably be considered for ELT (Bernal, 2010).

### INR

A number of studies illustrating the time course of daily INR values nicely show that a single INR level at the onset of HE (i.e., the overt manifestation of ALF) do not necessarily mirror the severity of liver damage and prognosis. This is particularly true for hyperacute ALF courses such as in acetaminophen or amanita toxicity (Escudié et al., 2007; Schmidt and Larsen, 2007). It is particularly in the subacute ALF setting that sustained elevations of INR become important for decision-making.

### Factor V and Clichy-Villejuif criteria

Early investigations of factor V in patients with fulminant hepatitis B have proven this marker to be an independent predictor of survival (Bernuau et al., 1986). Factor V, HE, and patients' age were later incorporated into the so called Clichy-Villejuif criteria which have been established particularly in France (Bismuth et al., 1995), but also form part of the German transplantation law (Richtlinien Organtransplantation, 2011). An unfavorable outcome can be expected in >90% of cases with HE plus a factor V level less than 20% of normal (in patients younger than 30 years of age) or less than 30% of normal (in patients older than 30 years of age), respectively (Table 1) (Bismuth et al., 1995).

**Table 1:** **Clichy-Villejuif criteria for non-acetaminophen ALF**.

Hepatic encephalopathy AND
Factor V <20%, if <30 years of age OR
Factor V <30%, if >30 years of age

### Ammonia

In ALF, ammonia escapes hepatic metabolism, leading to high arterial ammonia concentrations. Clemmesen et al. showed that ALF patients who developed clinical signs of cerebral herniation (*n* = 14 out of 44) had higher arterial plasma ammonia levels compared to those who did not (230 vs. 118 μmol/L, *p* < 0.001) (Clemmesen et al., 1999). This finding was later validated in a group of 165 patients with ALF and HE. An arterial ammonia level greater than 100 μmol/L was an independent risk factor for severe HE (sensitivity 59%, specificity 78%) and intracranial hypertension (sensitivity 73%, specificity 44%). The combination of arterial ammonia with the Model of End-stage Liver Disease (MELD) further increased specificity in prediction of HE (Bernal et al., 2007).

### Phosphate

Schmidt and Dalhoff published data on serum phosphate levels in 95 patients with severe acetaminophen poisoning. Phosphate concentrations were significantly higher in nonsurvivors than in survivors 48 h after overdose. A threshold phosphate concentration of 1.2 mmol/L at 48–96 h after overdose specifically and sensitively identified a group of patients with very little chance of spontaneous survival. The phosphate criteria had higher predictive values than the King's College criteria (KCC). The authors proposed that hyperphosphatemia is caused by renal dysfunction in the absence of hepatic regeneration, whereas the latter appears to be associated with lowering of serum phosphate. Possible limitations of this study were the relatively low numbers of patients with HE (*n* = 30) and a fatal outcome (*n* = 16) (Schmidt and Dalhoff, 2002).

### King's college criteria

The most commonly used prediction models are the KCC that were initially based on a retrospective study on 588 patients with ALF managed medically during 1973–1985. The model was validated in an independent cohort of 175 ALF patients treated between 1986 and 1987. The KCC distinguish between acetaminophen and non-acetaminophen-induced ALF and include pH, HE, INR, creatinine, etiology, bilirubin, age, and acuity of symptom onset (Table 2) (O'Grady et al., 1989). Several case series and meta-analyses have confirmed that KCC have clinically acceptable specificity of 80–90%, with survival without transplantation in patients meeting criteria of less than 15%. However, sensitivity of KCC has been reported to be as low as 60–70%, indicating that KCC may fail to detect patients facing a fatal outcome without ELT (Anand et al., 1997; Hadem et al., 2008; Bernal et al., 2010; Craig et al., 2010; McPhail et al., 2010). Despite this draw-back, KCC form the basis for ELT registration in many countries (Neuberger et al., 2008; Bernal et al., 2010; Richtlinien Organtransplantation, 2011).

**Table 2:** **King's college criteria**.

**ACETAMINOPHEN-INDUCED ALF**
Arterial pH <7.3 (regardless of HE)
OR all 3 of the following
– INR >6.5
– Creatinine >300 μmol/l
– HE grade 3–4
**NON-ACETAMINOPHEN-INDUCED ALF**
INR >6.5 (regardless of HE)
OR 3 of 5 of the following (regardless of HE)
– Age <10 or >40 years
– Etiology: indeterminate, drug-induced
– Time interval icterus to encephalopathy > 7 days
– INR >3.5
– Bilirubin >300 μmol/l

### Model of end-stage liver disease (MELD) score

Originally developed for allocation in patients with chronic liver disease, the MELD score has been evaluated as prognostic marker in ALF. Yantorno et al. investigated 64 adult ALF patients of mixed etiology and found MELD to provide better predictive value than KCC in a Cox model, defining a MELD of 30 as prognostic cut-off (Yantorno et al., 2007).

Another study examined MELD in 124 patients with acetaminophen-induced ALF. A threshold MELD score of 33 on the day after the onset of HE had a sensitivity of 60%, and a specificity of 69% in predicting death. However, the discriminative power of MELD score was not superior to that of INR alone or of the KCC (Schmidt and Larsen, 2007). Katoonizadeh et al. evaluated 99 patients with non-acetaminophen-induced ALF and found the best MELD cut-off to be >35 (sensitivity 86%, specificity 75%). Again, MELD was not superior to KCC (Katoonizadeh et al., 2007). A retrospective observational study on 134 German ALF patients of mixed etiology showed MELD to be an independent prognostic factor with a median MELD of 33 in the fatal prognostic group (Canbay et al., 2009). Another retrospective study on 102 ALF patients of mixed etiology from northern Germany demonstrated that MELD-based outcome prediction was comparable to that by KCC (Hadem et al., 2008). Finally, a modification of classic MELD by substituting the level of the M65 epitope of cytokeratin-18 (CK 18) for bilirubin, significantly improved the positive predictive value in a cohort of mainly 68 acetaminophen-induced ALF (Bechmann et al., 2010). The M65 epitope of CK 18 is exposed on all intact and fragmented CK18 variants released from destroyed hepatocytes, has been studied in several chronic liver disease entities (Bantel et al., 2001), and might thus allow a better quantification of liver damage in ALF (Volkmann et al., 2008).

### Bilirubin-Lactate-Etiology (BiLE) score

Bilirubin-Lactate-Etiology (BiLE) score was empirically developed based on 102 ALF patients of mixed etiology (predominantly indeterminate ALF) from northern Germany and found to be slightly superior to KCC, MELD, and SAPS-III in predicting death or need of ELT with a sensitivity of 79% and a specificity of 84%. BiLE score is calculated by adding bilirubin and lactate with an etiology-specific summand (Table 3) (Hadem et al., 2008). In an external retrospective evaluation of BiLE in 422 consecutive ALF patients from the UK (57% with acetaminophen-induced ALF), BiLE lacked sensitivity (55%), but had a good specificity of 89% (Bernal et al., 2009). As with other models, BiLE score has therefore not yet proven superiority over established models such as KCC, and it appears premature to base transplant decisions on BiLE at this point of time.

**Table 3:** **Bilirubin Lactate Etiology (BiLE) score**.

BiLE score =
Bilirubin (μmol/l)/100
+ Lactate (mmol/l)
+ 4 [in case of indeterminate ALF, Budd-Chiari syndrome or phenprocoumon toxicity]
− 2 [in case of acetaminophen toxicity]
+ 0 [in case of any other ALF etiology]

### Recent advances in ALF outcome prediction

An interesting prognostic scoring model for non-acetaminophen ALF has been proposed by colleagues from Japan. Four parameters (unfavorable etiology, HE grade 3/4, presence of systemic inflammatory response syndrome, and ratio of total to direct bilirubin >2) were evaluated retrospectively in 80 patients, and prospectively validated in another 26 patients on days 1, 4, 8, and 15. The scoring model predicted 2-week survival rates with high sensitivity and specificity, when all four time points of scoring were taken into account. However, the earliest score on day 1 which is the one of greatest clinical interest tended to underestimate the probability of a fatal outcome (Miyake et al., 2005).

As in liver cirrhosis, there has been a growing interest in applying scores of multiple organ dysfunction to ALF patients. Sequential Organ Failure Assessment (SOFA) score is an ordinal variable that covers six organ systems and has been established as dynamic disease severity measurement tool in intensive care (Vincent et al., 1996). Craig et al. investigated SOFA in 138 acetaminophen overdoses (only 48% with HE) and found that SOFA provided superior outcome discrimination compared to MELD. The authors stated, however, that due to limited specificity, SOFA would not be capable of replacing KCC as definite listing criteria (Craig et al., 2012). Moreover, organ dysfunction as documented by SOFA, typically develops >72 h following ALF onset and therefore does probably not represent a very early predictive tool.

Angiopoietin-2 (Angpt2), a mediator of endothelial activation and capillary leakage, is known to be up-regulated in sepsis, but its role in ALF has just recently been investigated. Angpt2 serum levels were examined in 37 patients with ALF of mixed etiology and 20 healthy controls. Angpt2 revealed to be a predictor of the composite end-point of death or ELT and correlated strongly with surrogate markers of organ dysfunction. Immunohistological studies showed that Angpt2 was upregulated in ALF explants (Hadem et al., 2012). Angpt2 might therefore explain the regularly observed sepsis-like clinical picture of ALF patients and also serve as a marker of impending multiple organ failure.

## Acute liver failure—whom and when to transplant in clinical practice

Current criteria for selection of patients with ALF for ELT are far from being perfect (Renner, 2007). The ideal means for identification of patients who are likely to benefit from ELT still remains controversial (Bernal et al., 2010). A patient who would have survived with medical management, incorrectly transplanted will be subjected to unnecessary surgery, life-long immunosuppression, and an increased risk of death. Furthermore, a graft that could be used in a more appropriate candidate will be lost (Bernal, 2010). Decisions regarding ELT have to be made early-on during the course of ALF because of impending complications such as cerebral herniation or multiple organ failure. In a retrospective study from King's College Hospital of 310 ALF patients listed for ELT, the median time from listing to ELT was 1 day. However, 24% of listed patients did not undergo surgery, mainly because of death on the waiting list at a median of 2 days after listing (Bernal et al., 2009). Similar observations were made in the US ALF study cohort with a median time from listing to ELT of 3.5 days and a median time from admission to death of 5 days (Ostapowicz et al., 2002).

The difficult questions whom and when to perform ELT in an individual patient are best answered in a multiple step approach.

First, the focus is on ALF etiology and HE grade to ensure that the ELT candidate will have a grim prognosis without ELT. Other predictive markers such as acuity of symptom onset, sustained increase in INR, or persisting elevation of lactate after adequate fluid resuscitation can be incorporated in the decision. Sometimes, daily ultrasound monitoring of progressing liver dystrophy can complete the physician's judgment on which therapeutic path to follow. Established prognostic models such as the KCC and the Clichy Villejuif criteria are certainly of value. All these factors should rather be regarded as pieces of a mosaic than definite criteria. It is rather the rule than an exception that a clear-sighted decision has to rely on a certain amount of physician's gut feeling. For example, slow clinical deterioration, moderately elevated INR and undulating bilirubin levels in a young female with subacute indeterminate ALF should not deceive the team about her likely bad prognosis. On the other hand, a patient with acetaminophen-induced ALF and hyperacute clinical presentation may have a high spontaneous survival rate even though experiencing advanced stages of disease (Schmidt and Larsen, 2010). A useful summary of the UK criteria for registration as high-urgent ELT candidate has recently been published by Neuberger et al. (2008).

Second, potential contraindications against ELT have to be excluded. Among them are (1) advanced biological patient age, (2) pre-existing chronic comorbidities (major psychiatric disorders probably influencing patient's post-transplant compliance, heavy alcohol abuse, cardiopulmonary disorders), (3) unfavorable severity of pretransplant illness (reflected by high-dose vasopressor support or multiple organ dysfunction), and (4) secondary ALFs in the setting of severe sepsis or generalized ischemia (e.g., after cardiopulmonary resuscitation). Patients with acetaminophen-induced ALF are at high risk of developing pre-ELT complications. Additionally, ELT recipients >50 years face a doubled postoperative mortality (Bernal et al., 2009).

Third, the quality of the liver graft influences post-ELT outcome and has to be matched in each individual case. It is very demanding to find the right balance between risking delay of ELT until an ideal graft is available and early acceptance of a suboptimum graft that might be associated with a poor outcome (Bernal et al., 2009, 2010).

## Severe acute alcoholic hepatitis—a new candidate for emergency liver transplantation?

Mathurin et al. recently reported on 26 patients with a first episode of severe alcoholic hepatitis that were not responding to medical therapy and selected to receive ELT. Six-month survival was markedly improved compared with patients not receiving ELT (78% vs. 24%). Three out of 26 patients (12%) experienced alcohol relapse (Mathurin et al., 2011). As these patients were highly selected involving the whole primary care team and focusing on the patient's social background, it remains open, if a broader consideration of alcoholic hepatitis for ELT would result in similarly good outcomes. The current discussion about alcoholic hepatitis as potential candidate for ELT focuses on the issue of justice among liver graft recipients, possible biases in selecting suitable candidates, the 12% relapse rate, the rather arbitrarily defined 6-month abstinence rule, and the high rate of perioperative infectious complications (Brown, 2011; John and Chung, 2012; Moreno et al., 2012).

## Conclusion

In conclusion, selecting the ALF patient in need of ELT, determining the right point of time for wait-listing, and matching patient and liver graft remains challenging. This demands experience as well as a high grade of suspicion. Available prognostic parameters and models can be of help, but should be regarded as pieces of a mosaic rather than definite therapeutic algorithms. Proper management of ALF patients have now achieved an increase in overall survival rates from 40% without availability of ELT to over 80% post ELT (Ostapowicz et al., 2002; Lee et al., 2011).

### Conflict of interest statement

The authors declare that the research was conducted in the absence of any commercial or financial relationships that could be construed as a potential conflict of interest.
